# Pannexin 1 sustains the electrophysiological responsiveness of retinal ganglion cells

**DOI:** 10.1038/s41598-018-23894-2

**Published:** 2018-04-11

**Authors:** Galina Dvoriantchikova, Alexey Pronin, Sarah Kurtenbach, Abduqodir Toychiev, Tsung-Han Chou, Christopher W. Yee, Breanne Prindeville, Junior Tayou, Vittorio Porciatti, Botir T. Sagdullaev, Vladlen Z. Slepak, Valery I. Shestopalov

**Affiliations:** 1 Bascom Palmer Eye Institute, Department of Ophthalmology, University of Miami Miller School of Medicine, 900 NW 10 Ave., Miami, FL 33136 USA; 2 Department of Molecular and Cellular Pharmacology, University of Miami Miller School of Medicine, 1600 NW 10th Ave., Miami, FL 33136 USA; 3 Department of Ophthalmology, Weill Cornell Medical College, 156 William St., New York, NY 10038 USA; 4 Winifred Masterson Burke Medical Research Institute, New York, 785 Mamaroneck Ave., White Plains, NY 10605 USA; 5 Department of Cell Biology, University of Miami Miller School of Medicine, 1600 NW 10th Ave., Miami, FL 33136 USA; 6 Vavilov Institute for General Genetics, Gubkina Str. 3, Russian Academy of Sciences, Moscow, Russia; 7 Kharkevich Institute for Information Transmission Problems, Russian Academy of Sciences, Moscow, Russia

## Abstract

Pannexin 1 (Panx1) forms ATP-permeable membrane channels that play a key role in purinergic signaling in the nervous system in both normal and pathological conditions. In the retina, particularly high levels of Panx1 are found in retinal ganglion cells (RGCs), but the normal physiological function in these cells remains unclear. In this study, we used patch clamp recordings in the intact inner retina to show that evoked currents characteristic of Panx1 channel activity were detected only in RGCs, particularly in the OFF-type cells. The analysis of pattern electroretinogram (PERG) recordings indicated that Panx1 contributes to the electrical output of the retina. Consistently, PERG amplitudes were significantly impaired in the eyes with targeted ablation of the Panx1 gene in RGCs. Under ocular hypertension and ischemic conditions, however, high Panx1 activity permeated cell membranes and facilitated the selective loss of RGCs or stably transfected Neuro2A cells. Our results show that high expression of the Panx1 channel in RGCs is essential for visual function in the inner retina but makes these cells highly sensitive to mechanical and ischemic stresses. These findings are relevant to the pathophysiology of retinal disorders induced by increased intraocular pressure, such as glaucoma.

## Introduction

Pannexin 1 (Panx1) is a high-conductance voltage-gated channel that connects the intracellular and extracellular spaces in vertebrate tissues. Panx1 allows the passage of molecules up to 1 kDa between these compartments, including ions, amino acids, nucleotides and other metabolites^1^. Panx1 channels serve as one of the major conduits for ATP release^2^ and contribute to purinergic and adenosine signaling^3,4^. Extensive evidence has accumulated for the role of Panx1 in neuronal pathologies, such as epilepsy and autism^5,6^, ischemic and traumatic brain injuries^7,8^, post-ischemic glutamate toxicity^9^, pain^10^ and inflammatory diseases^11,12^. However, the understanding of the normal physiological function of Panx1 in the central nervous system (CNS) is uncertain.

Panx1 is widely expressed in the CNS, and its expression levels vary dramatically between distinct cell types^13,14^. In the developing and adult retina, the expression of Panx1 is high in horizontal cells and inner retinal neurons, particularly in retinal ganglion cells (RGCs)^13^, the output neurons of the retina that send visual information to the brain visual centers. Currently, there is a gap in our knowledge of the physiological role of Panx1 in RGCs. Physiological experiments using *in vivo* and *in vitro* microchip-mediated electroretinogram (ERG) recordings from the inner retina have shown reduced amplitudes of a- and b-waves under scotopic conditions in Panx1-null retinas^15^. These results suggested that Panx1 function in the retina may involve photoreceptor, bipolar cell, or RGC function; however, the data generated by this technique cannot be directly attributed to RGC function.

The activity of RGCs is assessed electrophysiologically by pattern electroretinograms (PERGs). This technique, first described by Riggs *et al*.^16^ and applied to RGCs by Maffei and Fiorentini^17^, has become an integral clinical diagnostic tool for glaucoma. The amplitude of PERG responses directly correlates with the functional status of RGCs in rodents and humans^18^ and is highly sensitive to injuries induced in these neurons, including elevated IOP-induced injury. Reduced responses reflect the dysfunction and eventual loss of RGCs, which is the cause of blindness in glaucoma^19,20^. A pathological decrease in the amplitude of PERG responses is typically observed in glaucomatous and ischemic optic neuropathies^19–21^ and correlates with the loss or dysfunction of RGCs. Voltage-gated Na^+^ channels that produce action potentials serve as generators of the PERG signal in the retina^22^. However, whether other voltage-gated channels, particularly Panx1, also contribute to the PERG responses remains unknown.

Panx1 activation can be regulated by extracellular ATP (eATP) either directly^23,24^ or via purinergic P2 receptors^3^. In pathological settings, the release of eATP into the extracellular space from injured or dying cells represents a “danger signal”, typically observed in traumatic, ischemic, and inflammatory CNS disorders^25,26^, including ischemic stroke and intraocular pressure (IOP)-induced glaucoma^27,28^. Therefore, dysregulated Panx1 activity can be linked to reduced RGC survival.

In this study, we analyzed the pattern of Panx1 expression and activity in the adult mouse retina and explored the intriguing possibility that the Panx1 channel, which is abundant in RGCs, contributes to PERG responses. We also compared the channel activity among functionally distinct RGCs and obtained additional evidence that RGCs with high Panx1 expression and activity are prone to selective loss in ischemic and glaucomatous pathologies.

## Results

### RGCs have the highest level of Panx1 expression in the inner retina

**Figure 1:**
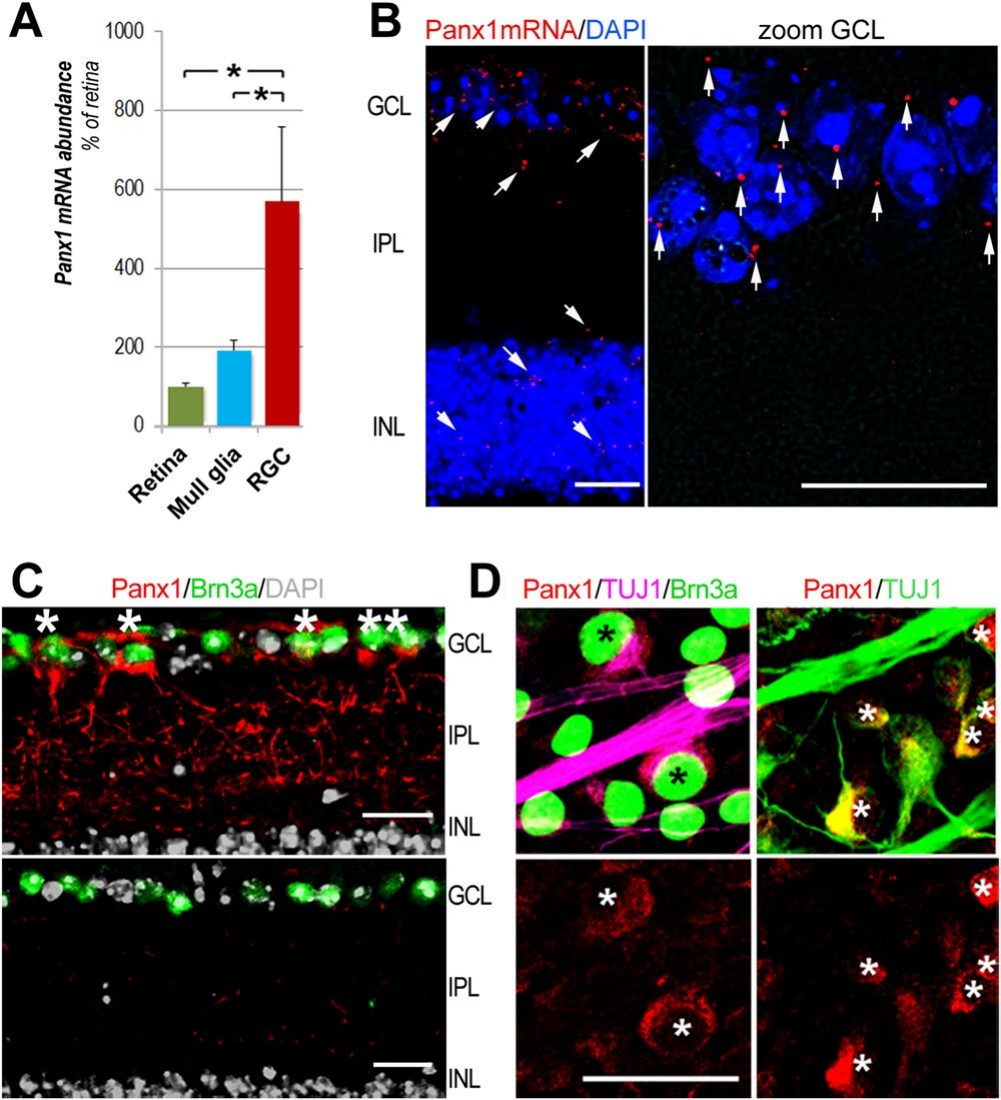
RGCs have the highest levels of Panx1 expression in the retina. (**A**) Real -time PCR in purified primary cells shows significant enrichment of Panx1 in RGC (red bar) vs. whole retina (green bar) and Muller glia (Muller GL, blue bar), *P ≤ 0.05: n = 5, Student’s t-test; (**B**) Representative micrographs of *in situ* RNA hybridization of Panx1 transcripts (red puncta indicated by arrows on the insert) using RNAscope technique. Insert (zoom, right panel) shows Panx1 transcripts in magnified ganglion cell layer (GCL) region, where RGCs are located; nuclei labeling: DAPI (blue); Scale bar, 25 µm. (**C**) Representative micrographs of immunostaining in retina sections: the highest level of Panx1 labeling (red) in the GCL co-localized with Brn3a-positive RGCs (green), as indicated by asterisks. The lower panel shows control staining in Panx1 knockout tissue. Scale bar, 25 µm. (**D**) Representative retinal flat-mounts co-immunostained for Panx1, and RGCs markers TUJ1 (magenta), and Brn3A (green). The level of Panx1 labeling (red) varied significantly among RGCs with higher than average levels detected in about one-third of all Brn3A-positive neurons (asterisks). The remaining RGCs (Brn3a-positive, no asterisks) showed significantly lower levels of the Panx1 protein. Scale bar, 25 µm.

The ganglion cell layer (GCL) contains a mixed population of RGCs, amacrine cells (ACs), and glial cells, all of which express different levels of Panx1^13,29^. Previous reports have localized Panx1 to the ganglion cells, bipolar cells^15^, dendrites and axonal processes of horizontal cells in the fish^30^ and mouse^13^ retina. To analyze cellular expression of Panx1 in the inner retina, we first examined gene expression in pan-purified retinal cells by quantitative RT-PCR. Our results indicate that the relative abundance of the Panx1 transcript in RGCs was approximately 6-fold higher than in the whole retina and 3-fold higher than in Muller glia (Fig. 1A). Consistently, quantitative *in situ* RNA hybridization using the RNAscope technique showed dramatic enrichment of Panx1 transcript labeling in the GCL (Fig. 1B). Next, to validate these data at the protein level, we performed immunostaining in retinal whole mounts and cross-sectional slices. Consistent with the gene expression data, the most intense Panx1-specific labeling was also observed in the GCL (Fig. 1C,D). A more detailed examination of retinal slices and whole mounts showed that Panx1 co-localized with tubulin βIII or Brn3a-positive cells (i.e., RGCs). This analysis also revealed striking heterogeneity in the intensity of individual cell labeling. In general, less than half of Brn3a- or tubulin βIII-positive cells showed high levels of Panx1 immunoreactivity (marked with asterisks, Fig. 1C,D), whereas the majority of RGCs showed significantly lower levels of labeling.

### Robust Panx1-mediated currents are detected only in RGCs

**Figure 2:**
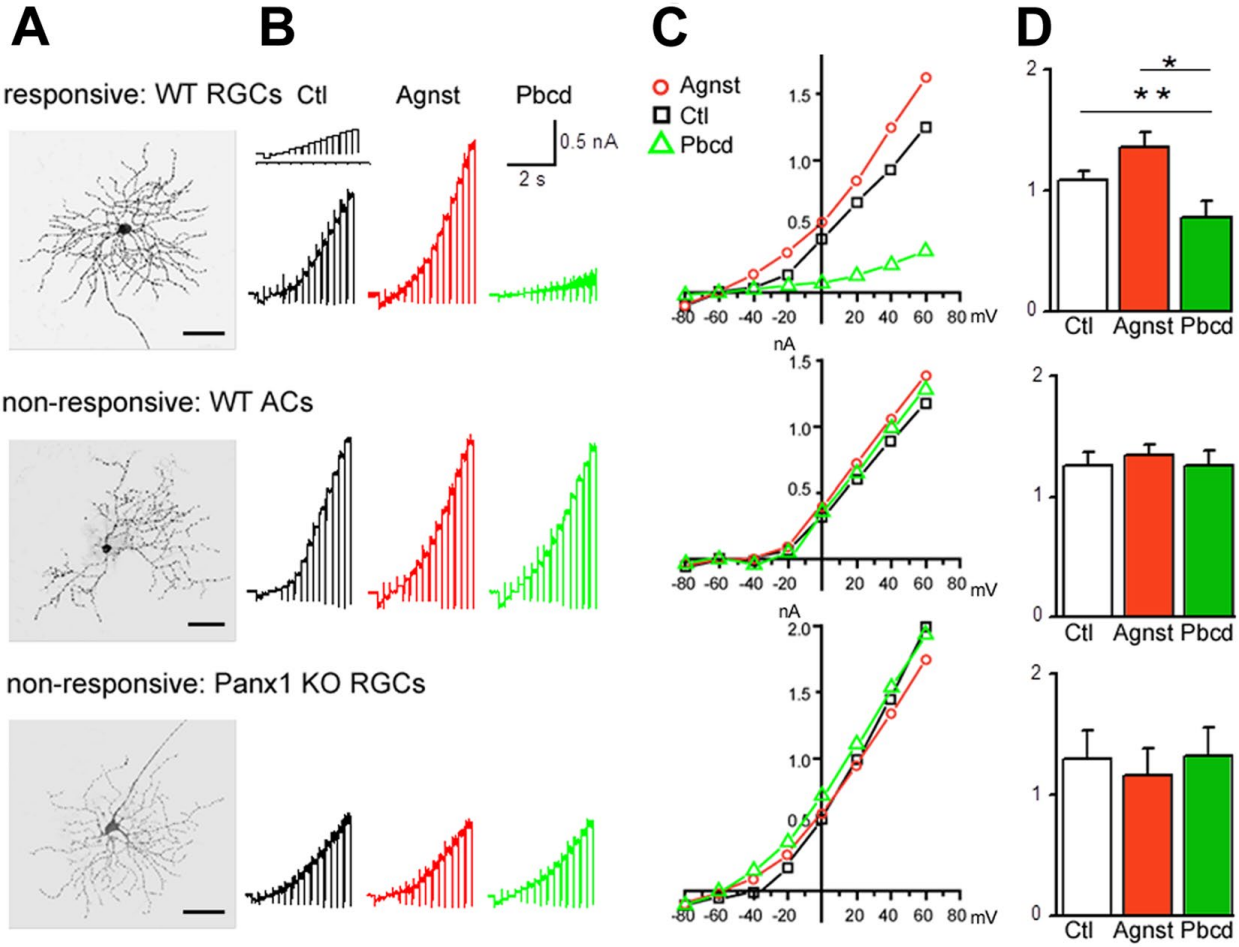
Voltage patch clamp recordings of Panx1 currents from inner retinal neurons. Activation of Panx1 currents in individually patched neurons in the GCL was triggered by applying depolarizing voltage steps (depicted in detail in Fig. 6C). (**A**) Representative micrographs of morphometric cell type identification, reconstructed from confocal z-stack datasets of Alexa-568 labeled RGCs. Neuron phenotyping was performed by the presence of an axon (arrows, left panels). Scale bar, 25 µm. (**B**) Representative current responses to the applied pulse protocol. Cells were treated with a Panx1 agonist cocktail (Agnst, 3 mM ATP, 20 mM KCl) or antagonist (Pbcd, 300 µM probenecid. (**C**) Representative traces of current-voltage relationship in single cell recordings. Experimental data points were obtained by plotting the current amplitude elicited by a given voltage step under the control condition, after application of the agonist or antagonist. (**D**) Quantitation graphs showing maximum currents (nA) evoked by a +80 mV voltage step in control and experimental conditions for each of the two RGC and one AC subpopulations with statistical analysis. Data are presented as means ± SE; n_responsive WT RGCs_ = 11; n_WT ACs_ = 7; n_Panx1 KO RGCs_ = 6. **P < 0.01; *P < 0.05; significance: Kruskal-Wallis test and Dunn’s Multiple Comparison test. Color coding: control bath solution, black; agonists, red; antagonist, green.

We used the whole-cell patch clamp technique to investigate whether high heterogeneity in the levels of Panx1 expression was reflected by a differential channel activity among neurons. We patched and characterized neurons in the GCL using a preconditioning voltage ramp paradigm^31^. Morphometric analysis using 3D confocal microscopy performed after each patch clamp recording allowed us to distinguish RGCs from non-projecting GCL neurons lacking an axon, most likely the displaced ACs (Fig. 2A), and group them for the type-specific analysis of responses. To detect the effects of known post-ischemic danger factors on Panx1 activity in primary RGCs, we perfused retinas with the “Panx1 agonist mix” (3 mM ATP, 20 mM K^+^, 0.5 mM Ca^2+^). Since the contribution of other channels can be considerable, we used experimental conditions to minimize it, such as a pipette solution containing Cs and TEA to block potassium currents, Q314 to block sodium currents and gluconate to eliminate some of the chloride current. In these conditions, the main sources of chloride currents are GABAergic and Glycinergic receptors, but Panx1 currents become detectable as well^31^. The analysis of GCL neurons in the WT retinas showed that the outward-rectifying currents were 1.08 ± 0.083 nA (n = 11) and were sensitive to probenecid, which is characteristic for Panx1 channels. These currents were only detected in the RGCs but not in the ACs (n = 7) (Fig. 2B,C). Among RGCs, we detected high heterogeneity in the levels of Panx1 activity, where cells showed either robust (n = 11) or muted (n = 6, data not shown) responses or a total lack of a response (n = 3) to the Panx1-specific activation paradigm (Fig. 2B–D, red traces). As expected, no response was detected in Panx1^−/−^ RGCs (n = 11, Fig. 2, lower panels) or Panx1^−/−^ ACs (n = 9, data not shown). On average, the agonist mix increased the outward currents by 25% (to 1.36 ± 0.119 nA) in all robustly responding RGCs, but the effect was not statistically significant in unresponsive cells. Application of the Panx1 channel blocker, probenecid (300 µM), significantly reduced the amplitude of the outward currents from 1.08 ± 0.083 nA to 0.773 ± 0.139 nA (Fig. 2B,C, green traces), contributing to approximately 30% of total current in the responsive cells. To further confirm the involvement of the Panx1 channel, we also applied other Panx1-selective blockers, including mefloquine (MFQ, 50 nM), carbenoxolone (CBX, 25 µM), and ^10^panx peptide (100 µM), which had similar suppressive effects on responsive cells (data not shown), indicating that these currents were, indeed, mediated by Panx1.

**Figure 3:**
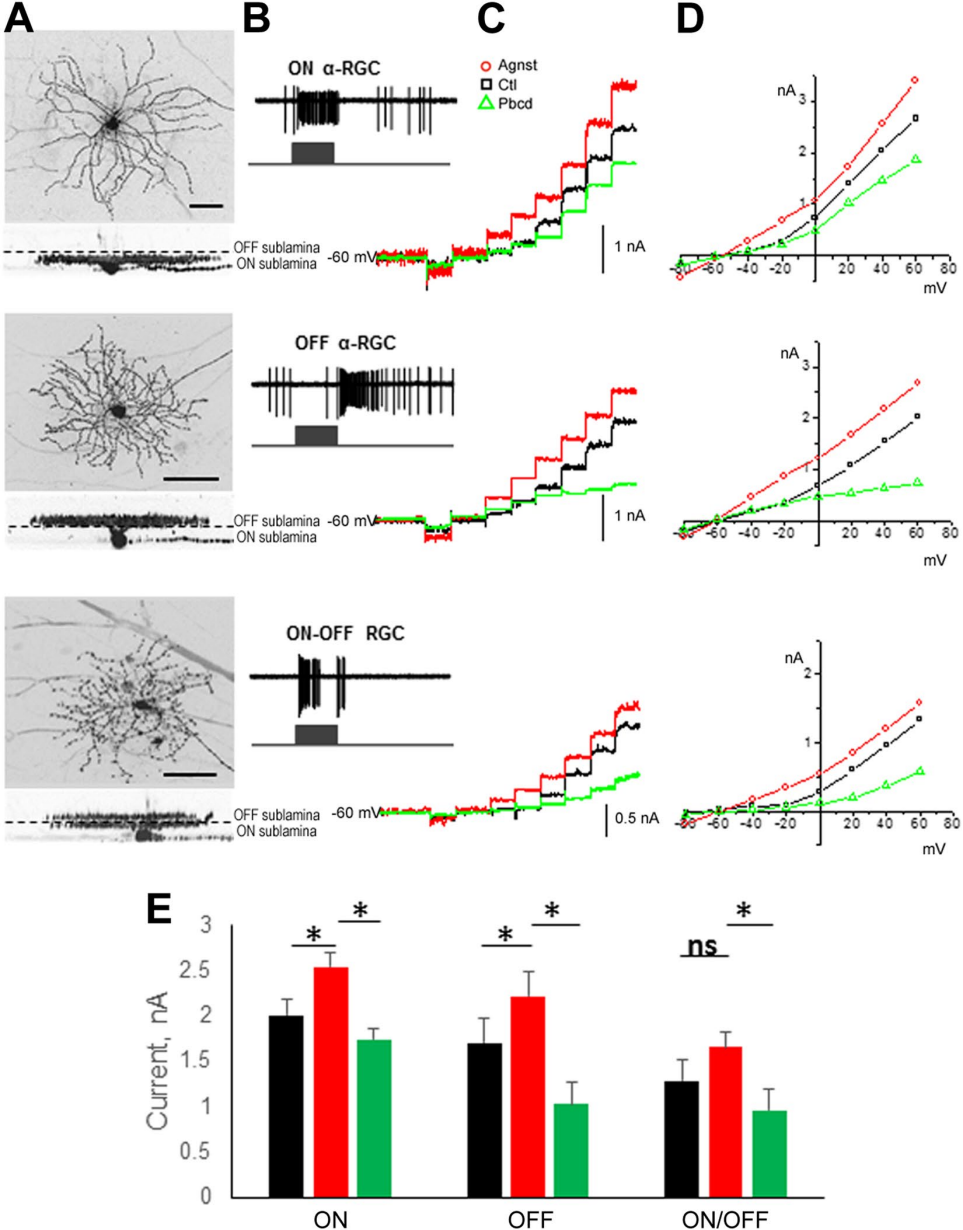
Panx1 channel activity in three functional subtypes of RGCs. (**A**) RGCs morphological types identification, performed by 3D confocal reconstruction of cells labeled with Alexa-568 dye; RGC types were defined by stratification of their dendritic tree in ON and/or OFF sublamina of the inner plexiform layer. Scale bar, 25 µm. (**B**) Functional typing of RGCs by spiking activity in response to the light stimulus. (**C**) Pannexin1 channel-activated currents in whole-cell mode in response to the depolarizing voltage steps from −80 to 60 mV with holding potential of −60 mV in control solution (black), with Panx1 agonist cocktail (Agnst, 20 mM K^+ ^0.1 mM ATP, red) or Pbcd (300 μM, green) conditions. (**D**) Representative I/V relationship for ON (n = 20), OFF (n = 20) and ON/OFF (n = 11) RGCs. (**E**) Mean values for the Panx1 channel currents; significance: Kruskal-Wallis test and Dunn’s Multiple Comparison test *P < 0.05, ns, non-significant color coding: control bath solution, black; agonists, red; antagonist, green.

To test whether such heterogeneity correlated with the major functional subtypes of RGCs, we performed additional patch clamp experiments where we specifically targeted RGCs. All recorded RGCs were divided into three subtypes, OFF-, ON- and ON/OFF-type RGCs, according to their light responses and dendritic tree projection into the inner plexiform layer (IPL) sublaminae (Fig. 3). All RGC types showed a statistically significant induction of the transmembrane current by the agonists, suggesting that the bulk of this response was Panx1-specific (Fig. 3E). However, only OFF-type RGCs showed significant inhibition of this current by probenecid, suggesting that the OFF-type subpopulation included the RGCs with the highest Panx1 channel activity in the inner retina.

Consistent with the immunohistochemistry results, the patch clamp experiments demonstrated heterogeneity in the levels of Panx1 expression and corresponding channel activity within the RGC population. To investigate the physiological relevance of such heterogeneity, we performed a series of behavioral and physiological experiments and cell survival tests.

### Panx1 contributes to the PERG response

To examine whether Panx1 is essential for normal retina function, we first assessed visual acuity using the visually guided behavioral head-turning test. The optokinetic response measurements performed at standard lane contrast settings revealed no significant differences between WT and Panx1^−/−^ mice (Fig. S1). Next, we examined the potential involvement of Panx1 in the function of the outer vs. inner retina circuits. The outer retina function in these mice was assessed with flash ERG (FERG) recordings and did not reveal any differences between WT and Panx1^−/−^ eyes (data not shown).

**Figure 4:**
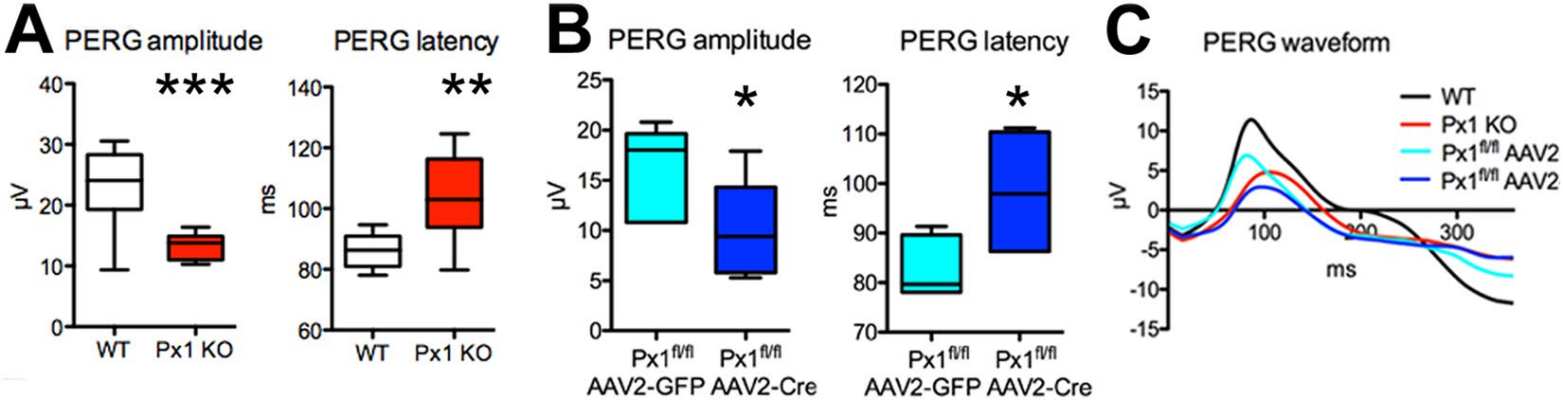
Panx1 deficiency results in changes in RGC function. PERG responses were compared between C57Bl/6 wild-type (WT) and Panx1^−/−^ mice as described in Materials and Methods. (**A**) Box graphs of PERG responses in germline Panx1^−/−^ (Px1 KO). Left panel: PERG amplitude; right panel: PERG latency. n_WT_ = 12; n_KO_ = 12. (**B**) PERG response in RGCs with conditional Panx1 gene ablation. The recordings were performed 8 weeks after infection of the AAV2-GFP-Cre construct via intravitreal injection; n = 5. (**C**) Averaged waveforms of responses recorded from WT (white boxes), germline Panx1^−/−^ (Px1 KO, red), AAV2-EGFP-injected Panx1^fl/fl^ controls (AAV2-GFP, light blue) and conditional AAV2-Cre-EGFP –induced Panx1^RGC−/−^ (AAV2-Cre, dark blue) eyes. ***P < 0.001; **P < 0.01; significance: Mann Whitney test.

Inner retina function was examined by recording the PERG, which allowed for the assessment of RGC function^32^. In contrast to the FERG results, PERG recordings have revealed significant differences between Panx1^−/−^ and WT mice. The PERG amplitude was 43.1 ± 6.9% lower in the Panx1^−/−^ retinas, while the latency of the PERG response was 20.2 ± 2.7% longer than that in the WT retinas (Fig. 4A, red waveform in C). The phenotype in the germline Panx1 knockout may be influenced by compensatory gene activation. To avoid this and test whether Panx1 channels endogenous to RGCs contribute to the PERG response, we analyzed the PERG in mice where the *Panx1* gene was selectively inactivated in RGCs. This conditional gene ablation was induced in adult animals by an intravitreal AAV2-Cre injection, an approach that was previously demonstrated to consistently generate targeted RGC-specific gene deletions^33^. We unilaterally injected Panx1^fl/fl^ mice with the AAV2-Cre-EGFP virus; contralateral control eyes received the control AAV2-EGFP construct. The control and experimental groups showed an average infection rate of 97.1 ± 5.6% for the AAV2-Cre-EGFP construct and 91.3 ± 4.5% for the control AAV2-EGFP construct. Sequential recordings, performed at 4, 6, and 8 weeks post-injection, showed a gradual decrease in the PERG amplitude in AAV2-Cre-EGFP –infected eyes. The reduction became statistically significant at 8 weeks when the mean amplitude dropped by 45.6 ± 15.5% relative to that in control eyes (Fig. 4B and blue waveform in C). An increase in the PERG latency was also observed at 6 weeks after injection with the AAV2-Cre virus but it only became statistically significant at 8 weeks (Fig. 4B). The dynamics of these parameters showed that the PERG amplitude consistently decreased, whereas the PERG latency increased relative to that in the control eye only between 4 and 8 weeks following AAV2-Cre-EGFP injection.

### Neurons are more resistant to IOP-induced injury in retinas with Panx1 inactivation

**Figure 5:**
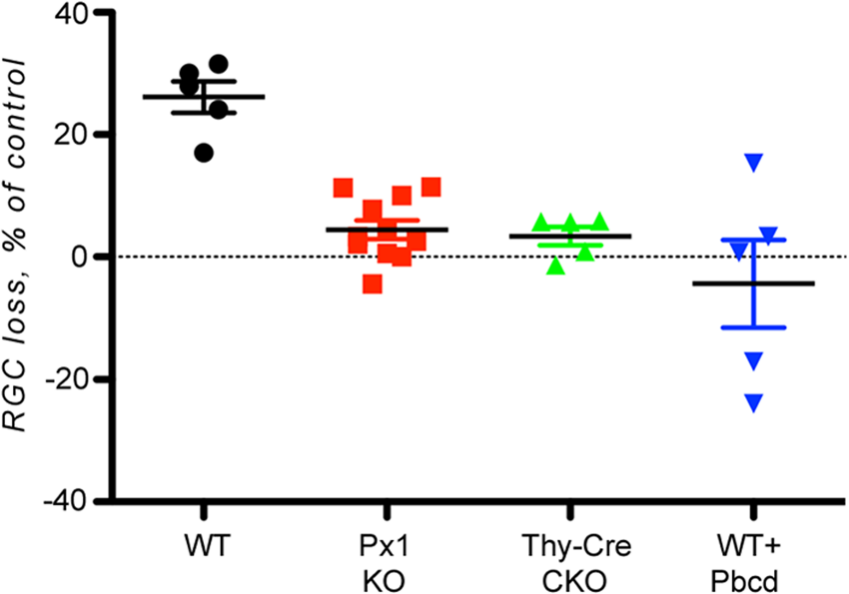
Panx1 inactivation protects RGCs in ischemia-reperfusion. The rate of survival following ischemia-reperfusion injury in the retinas with germline (Px1 KO, red, n = 11) and neuron-specific (Px1-CKO, green, n = 5) Panx1 ablation, WT C57Bl/6 control retinas (WT, black, n = 10) and wild type retinas from the probenecid-treated mice (WT + Pbcd, blue, n = 5). The data show percentage of NeuN-positive cells left after treatment relative to control. Data show values determined for individual animals; the bars represent means ± SD; **P < 0.01; *P < 0.05; significance: one-way ANOVA and Tukey test for multiple comparisons.

To test whether the resistance of RGCs in Panx1^−/−^ mice to hypertension-induced ischemia is linked to Panx1 channel activity, we compared RGC loss in zygotic knockouts, conditional neuronal knockouts and in WT retinas treated with 2.0 mM probenecid. We challenged the retina with transient ischemia-reperfusion (IR) injury induced by a 45-minute-long IOP elevation. The analysis of the NeuN-positive cell density in the GCL at seven days post-injury showed that the mean cell loss was reduced 5.9-fold and 7.8-fold in germline Panx1^−/−^ mice and conditional neuron-specific Thy1-Cre/Panx1^fl/fl^ mice (to 4.5 ± 5.1% and 3.4 ± 3.4%, respectively), compared to the mean loss of 26.4 ± 4.9%, observed in WT C57BL/6 retinas (Fig. 5). The density of NeuN-positive cells in the probenecid-treated WT mice at seven days post-injury was not significantly different from either knockout strain (4.4 ± 16%). These results indicate that the channel activity is pivotal for Panx1 neurotoxicity.

Next, we determined whether the recently discovered “passenger” Casp11^del^ (Casp 4 in humans) mutation that inactivates the caspase 11 protease in our Panx1^−/−^ strain^34^ affects neuronal survival in IR injury. We challenged the retinas with IR and compared the RGC-loss rates in the animals with the germline Casp11 knockout (Casp11^−/−^) and those in a different Panx1 knockout (Panx1^−/−^/Casp11^+/+^) strain lacking the Casp11^del^ passenger mutation, developed by Dixit^35^. In these experiments, we applied isoflurane gas instead of ketamine/xylazine anesthesia to take advantage of a resulting increased (from 26% to approximately 65%) post-IR injury RGC loss rate, which would allow us to detect even minor differences in protection. Casp11^−/−^ mice showed a 72.7 ± 2.9% loss of NeuN-positive cells that was not significantly different from the loss observed in the WT (64.7 ± 5.2%) retina (Supplement Fig. S2). In contrast, Panx1^−/−^/Casp11^+/+^ mice showed 41.1 ± 8.9% loss, which was a 31.7% reduction relative to the loss in WT control mice. Combined, these data demonstrate that inactivation of Casp11 does not contribute to RGC protection.

### Modeling Panx1-mediated responses in stable Neuro2a (N2a) cell lines

**Figure 6:**
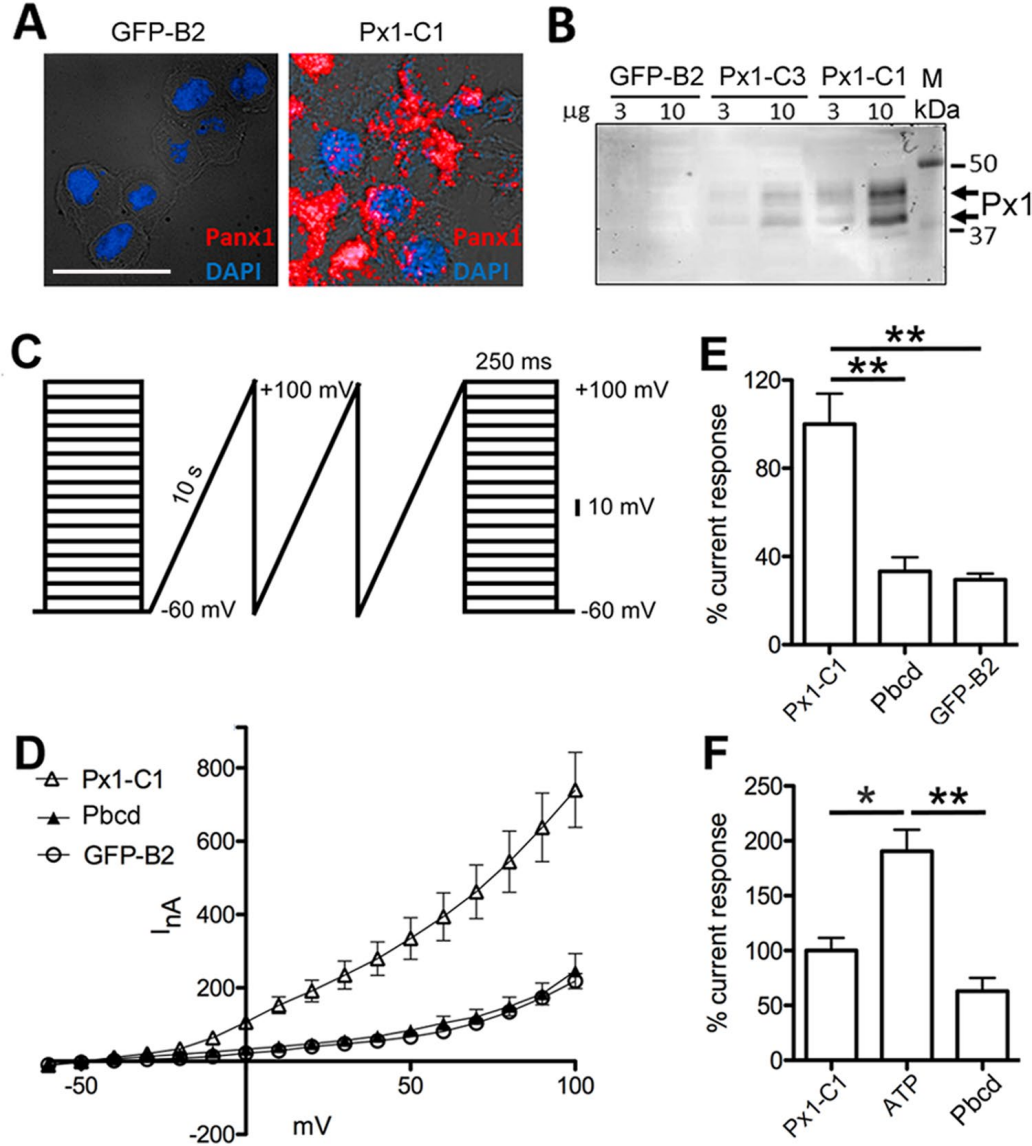
Panx1 forms functional channels in stably transfected N2a clones. (**A**) mRNA *in situ* hybridization analysis in stable N2a cell lines with (Px1-C1) and without (GFP-B2) Panx1 expression. The cells were labeled with the Panx1-specific fluorescent probe (red) to visualize transcripts and DAPI (blue) to visualize nuclei. Scale bar, 25 µm. (**B**) Western blot analysis of Panx1 protein expression levels in cellular extracts from three stable N2a clones, expressing either EGFP alone (GFP-B2) or both Panx1 and EGFP (Px1-C1 and Px1-C3). Loading: 3 or 10 µg of total protein per lane, as indicated. Panx1 (Px1) isoforms formed characteristic multiple bands 43–47 kDa, as indicated by the arrows. (**C**) Preconditioning (depolarizing) voltage ramp protocol to stimulate Panx1 channel activity, applied during electrophysiologial recordings. (**D**) I/V relation from N2a-Panx1-C1 (Px1-C1; n = 7) with and without 1.0 mM probenecid (Pbcd; n = 7) application; N2a-EGFP-B2 (GFP-B2; n = 8) expressing cells. (**E**) Data analyses and quantitation of evoked membrane currents response elicited at +100 mV from D. (**F**) Quantitation of evoked membrane currents response elicited at +100 mV from N2a-Panx1-C1 cells (n = 7) treated with 50 μM ATP (n = 7) and 300 μM Pbcd (n = 5); I/V relation graph is not shown. Data were normalized to N2a-Panx1-C1 membrane currents responses at +100 mV, set to 100%. Each bar represents mean ± SEM; **P < 0.01; *P < 0.05; significance: one-way ANOVA and Tukey test for multiple comparisons.

**Figure 7:**
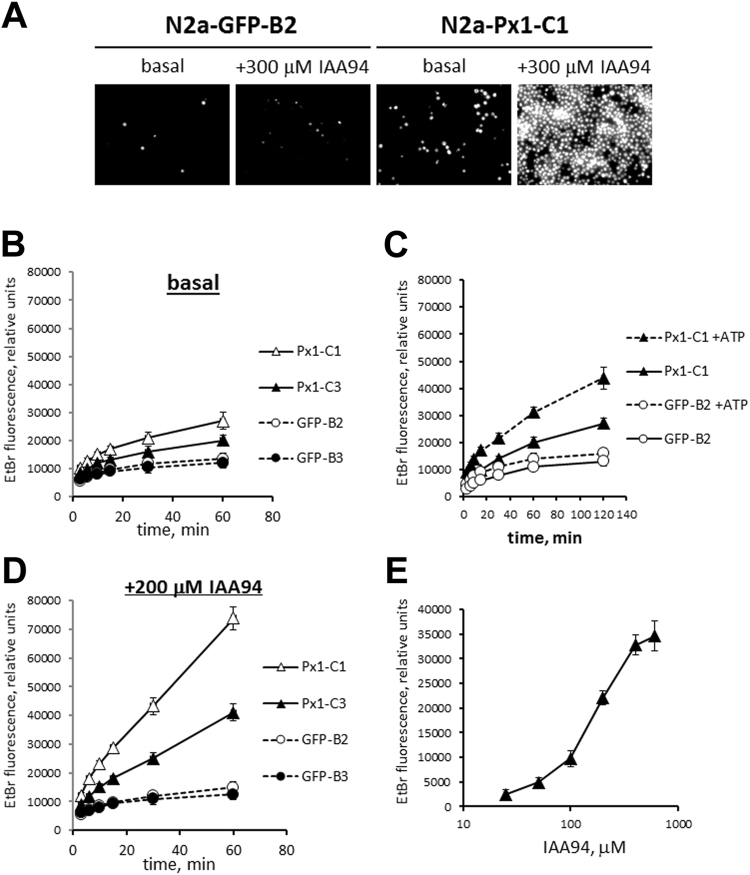
Overexpression of Panx1 increased N2a plasma membrane permeability to small molecules. (**A**) Ethidium bromide (EtBr) uptake by stably transfected N2a clones after 1 h incubation with and without IAA94 agonist. Cells were imaged by fluorescent microscopy at low (10x) magnification. (**B**–**E**) Time courses of EtBr uptake with or without pharmacological treatments. (**B**) Time course without any treatments (baseline). (**C**) Time course for EtBr uptake in the presence of 1.5 mM ATP. (**D**) Time course for EtBr uptake in the presence of 200 µM IAA94. (**E**) Dose-response curve for IAA94 in N2a-Panx1-C1 cells. Measurements were taken 30 min after application of IAA94; the basal value of EtBr uptake in the absence of IAA94 was subtracted from the recorded values; n = 9 per condition/time point.

**Figure 8:**
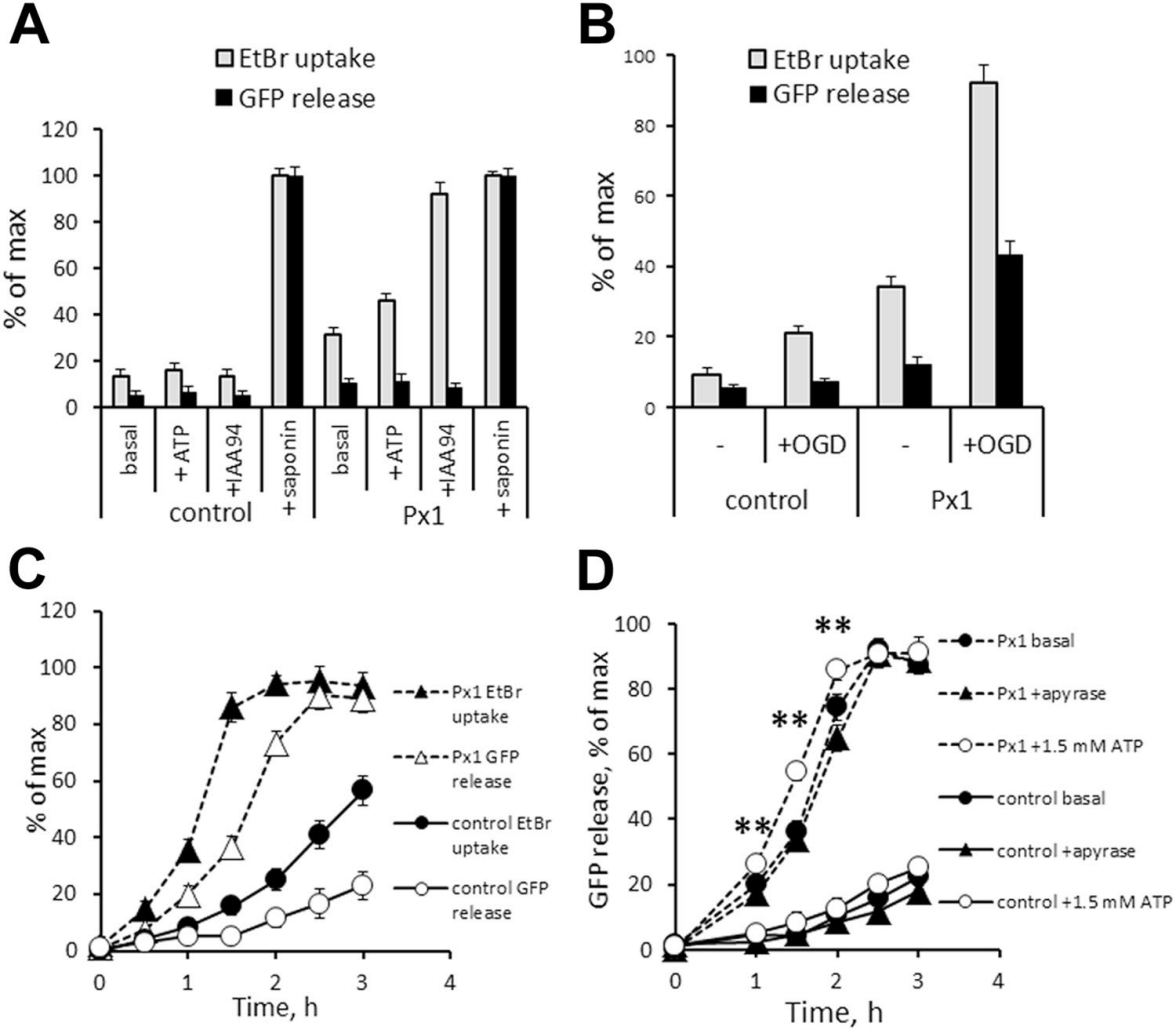
Overexpression of Panx1 increases susceptibility of N2a cells to OGD injury. Plasma membrane permeability to extracellular EtBr (uptake) and intercellular EGFP (release) in stably transfected N2a lines was quantified under the following conditons. (**A**) Normoxia for 2 h with or without 1.5 mM ATP or 0.2 mM IAA94. (**B**) Normoxia or OGD for 1.5 h. EGFP release under OGD conditions indicate cells death by membrane breakdown. (**C**) Time course of EGFP release under OGD conditions. (**D**) EGFP release under the OGD conditions with or without apyrase or 1.5 mM ATP. Values are percentage of the maximum value observed after treatment with 0.03% saponin. **Time points with statistically significant (P < 0.01) increase in EGFP release by Panx1-C1 cells in the presence of ATP; significance calculated using one-way ANOVA and Tukey test for multiple comparisons. Error bars for most values are smaller than symbols, n = 9 per condition/time point.

To model responses to ischemia and danger signals in retinal cells with significant differences in Panx1 expression, we generated stable N2a cell lines with high and low levels of Panx1 expression. We selected two N2a lines expressing Panx1 (N2a-Panx1-C1 and N2a-Panx1-C3) and two control line expressing only EGFP (N2a-EGFP-B2 and N2a-EGFP-B3). Similar to untransfected N2a cells, N2a-EGFP-B2/B3 lines expressed very low levels of endogenous Panx1 gene, detectable only by RT-PCR (data not shown). The difference in Panx1 expression levels between the N2a-Panx1-C1 and N2a-Panx1-C3 cell lines was confirmed by RNAscope *in situ* hybridization and quantitative Western blot analyses, as shown in Fig. 6A,B. In contrast, Panx1 could not be detected by either technique in the non-transfected (WT) N2a or N2a-EGFP-B2/B3 control cells lines. Overexpression of Panx1 or EGFP in these lines did not affect cell morphology or viability under normoxic conditions. The plasma membrane integrity in these cell lines was also unaffected and remained impermeable to both small molecules, like ethidium bromide (EtBr, Fig. 7B) dye and large molecules, such as EGFP (Fig. 8A).

To confirm the functionality of the Panx1 channels in the N2a-Panx1-C1 line with the highest Panx1 expression level, we performed whole-cell voltage patch clamp recordings using the preconditioning depolarizing voltage ramp paradigm described earlier^31^ (Fig. 6C). Large exponentially increasing outward rectifying currents were detected in N2a-Panx1-C1 but not control N2a-EGFP-B2 cells. The residual currents in the control cells were equivalent to the responses in the N2a-Panx1-C1 cells treated with 1.0 mM probenecid, which decreased the evoked responses in these cells by 66.8% when held at a potential of +100 mV (P < 0.01, Fig. 6D). The current observed in the N2a-Panx1-C1 cells displayed the Panx1-characteristic I/V signature (Fig. 6E). The maximum current amplitudes at +100 mV were 740 ± 102 nA for N2a-Panx1-C1 cells (n = 7), 245 ± 47 nA (n = 7) for N2a-Panx1-C1 cells treated with probenecid, and 218 ± 20 nA (n = 8) for N2a-EGFP-B2 cells. Application of 500 µM ATP resulted in a 90.5% increase in the membrane current response from N2a-Panx1-C1 cells held at +100 mV (P < 0.05; n = 7), which was suppressed by 66.9% (P < 0.01; n = 4) with the probenecid treatment (Fig. 6F). Because our results confirmed the expression of functional Panx1 in stably transfected N2a-Panx1-C1 cells, these cells were used in further experiments.

### Panx1 agonists and ischemia induce plasma membrane permeability in N2a-Panx1-C cell lines

To study the effects of the Panx1 expression level on plasma membrane permeability, we compared EtBr dye uptake by N2a-Panx1-C1/C3 clones with the uptake by the control N2a-EGFP-B2/B3 clones. We found that the basal dye uptake in control N2a-EGFP-B2/B3 clones was very low relative to that in Panx1-overexpressing N2a-Panx1-C1 cells (Fig. 7A,B). The increase in EtBr uptake was proportional to the level of Panx1 expression, as demonstrated by a 2-fold difference between the N2a-Panx1-C1 and N2a-Panx1-C3 clones (Fig. 7D).

Next, we investigated the effects of several compounds known to influence Panx1 activity. We found that application of 1.5 mM ATP, a known Panx1 activator, had no effect on the dynamics of the EtBr uptake in control N2a-EGFP-B2 cells but induced a 2-fold increase in dye uptake by the N2a-Panx1-C1 clone after a 2-h incubation (Fig. 7C). An even higher degree of plasma membrane permeation was detected after application of IAA94, a compound initially described as a chloride channel inhibitor^36^. IAA94 stimulated EtBr dye uptake without affecting cell viability (Fig. 7A) and in a gene dose-dependent manner, i.e. directly proportional to the Panx1 expression level (Fig. 7D). This resulted in a maximal level of uptake after 2 h of treatment (Fig. 7E), which was similar to the uptake caused by the membrane-permeating non-ionic detergent saponin (Fig. 8A). The effects of both ATP and IAA94 were not observed in the N2a-EGFP-B2 control cells at any time and, thus, were specific to Panx1 activity.

We then tested whether the difference in Panx1 levels could explain the differential resistance of neurons to ischemia induced by oxygen-glucose deprivation (OGD). Relative to the basal uptake level, the permeability to EtBr of both control N2a-EGFP-B2 and N2a-Panx1-C1 cells after OGD exposure was approximately 2.5-fold higher (Fig. 8B). However, the N2a-Panx1-C1 cells demonstrated a 5- to 7-fold higher EtBr uptake relative to control at 1 and 2 h post-OGD, respectively. At 2 h after OGD exposure, the rate of dye uptake in Panx1-expressing cells was over 90% of the maximal level compared to only 20% in similarly treated control cells (Fig. 8B).

### High Panx1 expression sensitizes N2a cells to ischemic injury

To monitor changes in plasma membrane integrity, we performed time-lapse recordings of EGFP (a 27 kDa protein) released into the medium as a marker of cell death and membrane rupture. In parallel, we analyzed the membrane permeability to small molecules, detected as EtBr (a 400 Da dye) uptake. Time-lapse recordings indicated that under normoxic conditions, all of the cells remained impermeable to EGFP, even after treatment with ATP or the IAA94 compound. The application of IAA94 has increased membrane permeability to EtBr to 95% of maximal in a Panx1-dependent manner (Fig. 8A).

However, exposure to OGD for 30–90 min resulted in a robust EGFP release from N2a-Panx1-C1 cells, indicating plasma membrane breakdown and the loss of cell viability (Fig. 8B,C). Control N2a-EGFP-B2 cells showed rather limited EGFP release that became detectable only after prolonged 90–180 min exposure to OGD. The effects of Panx1 on cell permeability were significantly increased in the presence of eATP, which facilitated a statistically significant 20–50% elevation in both EtBr uptake and GFP release in the N2a-Panx1-C1 cells at 1–2 h of OGD treatment (Fig. 8A,D). At 2 h after OGD treatment with eATP exposure, the cell death rate in control cultures averaged 10%, as compared to 74% in Panx1-expressing cultures (Fig. 8C). Overall, throughout the duration of the experiments, eATP treatment increased EGFP release in N2a-Panx1-C1 cultures by 55–70% relative to that in control cells (Fig. 8D), an indication of a more profound and/or accelerated membrane breakdown. This increase was induced specifically by eATP, since treatment with the ATP-hydrolyzing enzyme apyrase completely eliminated the effect (data not shown). Noteworthy, in cultures not treated with ATP, apyrase treatment had no effect on the rate of OGD-induced EGFP release. Thus, eATP exacerbated the OGD injury in a Panx1-dependent manner. Alternatively, cell survival using the annexin V/propidium iodide (PI) viability assay was used to further validate the Panx1-mediated effects on susceptibility of N2a cell to OGD injury. Only N2a-Panx1-C1 cells showed a significant 31.5 ± 5.5% reduction in the proportion of viable cells, paralleled by an equivalent increase in cells committed to death and positive for annexin V and/or PI labeling (Supplementary Fig. S3). In contrast to the EGFP release assay, quantification of the annexin V/PI staining was complicated by a considerable detachment of OGD-challenged N2a-Panx1-C1 cells, which were subsequently lost during the washing steps. Apparently, this resulted in a gross underestimation of dead cells observed in OGD-challenged cultures.

## Discussion

In this study, we investigated the function of the Panx1 channel in the normal and ischemic mouse retina and characterized its expression in different cell types. Using a targeted gene knockout approach, we found a correlation between high Panx1 expression, channel activity and an increased PERG amplitude in RGCs, which provided evidence for the essential role of Panx1 in normal RGCs. Patch clamp recordings revealed that increased levels of Panx1 activity were characteristic of distinct sub-populations of RGCs, particularly the OFF-type neurons. High levels of Panx1 expression are essential for the normal activity of functionally specialized RGCs, as they contribute to the electrical output needed to maintain PERG responsiveness. We also noticed the difference in agonist response curves among OFF and ON-OFF cells (Fig. 3D), which can possibly be explained by difference in the activity levels of Panx1 or its functional partners, NMDA receptors, which we demonstrated earlier in these sub-types^37,38^.

The PERG signal represents a light-adapted response of RGCs and is a sensitive index of overall RGC function. PERG is the most specific, non-invasive technique used for assessments of RGC functionality^18^. To the best of our knowledge, this is the first study to use PERG responses to examine the functional significance of Panx1 channels. The decrease in the PERG amplitude in Panx1-deficient mice suggests that RGCs enriched in Panx1 contribute to the response. More direct evidence of such involvement was obtained in AAV2-Cre-infected eyes of the Panx1^fl/fl^ animals, where RGC-selective transduction^39,40^ facilitated targeted Cre recombinase expression, resulting in a conditional Panx1 gene deletion. In these eyes, progressive depletion of the remaining Panx1 channels in RGCs resulted in a progressive decline in PERG amplitude. These data showed that the effect on the PERG response is RGC-specific and can be directly attributed to the ablation of the endogenous Panx1 channels. The selective effect of Panx1 deletion on the electrical excitability of RGCs is the first evidence that Panx1 channels are likely employed to increase the electrical output of these neurons. This result is consistent with a relative enrichment of this channel protein in RGCs, which was demonstrated using cell type-specific gene expression, mRNA and protein accumulation data (Fig. 1) and single-cell patch clamp recording (Figs 2–3). Importantly, the reduction in the PERG response in Panx1-deficient mice was not associated with a corresponding reduction in the photopic flash ERG b-wave. Both our study and the report by Kranz *et al*.^15^ showed that the photopic flash ERG (an outer retina response) is not altered in Panx1-deficient mice, which rules out a measurable role of Panx1 in pre-ganglionic retina function. The same study also attempted to test the possible roles of Panx1 in the retinal electrical output using microchip-mediated ERG recordings from the inner retina^15^; however, in contrast to our results, the data generated by this technique could not be directly attributed to RGC function. Also noteworthy, a recent report implicated Panx1 in the electrophysiology of the heart^41^, which is consistent with its proposed electrophysiological role in retinal neurons.

For the first time, we were able to determine that OFF-type cells possess the highest activity of Panx1 channels using single-cell patch clamp recordings. This new knowledge sheds light on the molecular constituents contributing to the functional specialization of RGCs, the output neurons consisting of approximately 30 functional subgroups^42^. Single-cell profiling experiments are required to identify molecular partners of Panx1 and the signaling pathways required for stimuli-dependent depolarization of these neurons.

The physiological mechanism by which Panx1 increases the electrical output in homeostatic RGCs currently remains unknown. The most studied pathway mediated by the Panx1 channel involves ATP release into the extracellular space. ATP and its degradation product, adenosine, participate in both paracrine and autocrine signaling, which facilitates activity of these output neurons^43,44^. Indeed, Panx1-mediated ATP release from RGCs^43^ and astrocytes^45^ was linked to neuronal activity in the central^46^ and peripheral nervous systems^47^, as well as in its regulation by the microglia^48^. Therefore, a synchronized activation of multiple Panx1 channels during RGC membrane depolarization may generate a local “puff” of eATP and a subsequent increase in adenosine, causing autocrine stimulation of P2X, other ATP-gated channels and A2A adenosine receptors.

The intrinsic responsiveness of Panx1 channels has previously been implicated in neurotoxicity, including neurotoxicity of RGCs, induced by mechanical or ischemic injuries^49–52^. In pathological conditions of elevated IOP, eATP and/or ischemia, high levels of Panx1 sensitized cells to death, while both ablation of the Panx1 gene or blockade of the Panx1 channel by probenecid provided protection *in vitro* and *in vivo* (Figs 5, 8). This result is important because it shows the following: a) Panx1 activity during the acute phase of IOP/eATP elevation-induced injury is a critical trigger for progressive RGC loss and b) transient suppression of the channel during acute injury is sufficient to provide significant protection. Furthermore, our dye uptake data in N2a cell cultures (Figs 7, 8) showed that such sensitization could cause selective dysfunction or death of Panx1-enriched N2a cells via membrane permeation and breakdown when exposed to ischemia and eATP. If the same is true for post-ischemic RGCs, this result suggests that the selective death of the Panx1-enriched RGCs as a feasible explanation of the early decrease in PERG amplitude typically observed in glaucoma and retinal ischemia^19,20^. Indeed, according to our AAV2-Cre conditional ablation data (Fig. 4), the Panx1-mediated loss of RGCs disrupted the PERG response in a similar fashion. Combined with a recent reports showing that distinct RGC subtypes that project dendrites into the OFF lamina are selectively affected in glaucoma^53,54^, our finding of a high Panx1 activity in OFF-type neurons suggests a role of Panx1-mediated toxicity in glaucoma RGC pathology.

The physiological mechanism facilitating Panx1-mediated neurotoxicity implicates pathological over-activation of the channel. What factors can contribute to such activation and synergistically sensitize neurons to death in the retina? A number of stressors and danger factors capable of activating the Panx1 channel are known to be present and/or increased in the retina upon challenge with IOP elevation, including: i) mechanical stress from the increased IOP^2^; ii) eATP, acting through purinergic P2X receptors^3^; iii) caspases 3/7, which irreversibly activate Panx1 by proteolytic cleavage^55^; iv) ischemic injury^7,49,56^; v) extracellular glutamate, which activates Panx1 via the NMDA receptor/Src kinase pathway^9^; and vi) other danger factors, such as high levels of extracellular K^+^, Zn2^+^ or β-amyloid^57–59^. In the current study, we examined the toxic effect of a combination of ischemia and ATP and utilized N2a cells engineered to over-express Panx1. Since electrophysiological responses to voltage ramps and to eATP were strikingly similar in Panx1-overexpressing N2a cells and in primary RGCs, we used the N2a data as a proxy for studies of RGC responses to ischemic stressors. We showed that this combination of stressors fully activated the Panx1 channels and selectively permeated and killed Panx1-enriched N2a cells. Indeed, our results show that elevated IOP, known to induce ATP release in the retina and optic nerve^27,43,45^, permeabilized and killed RGCs *in vivo* in a Panx1-dependent manner. This observation is in contrast to that observed in control N2a cells and non-RGC retinal neurons, such as displaced ACs. These cells, characterized by low levels of Panx1 expression and the lack of Panx1-specific transmembrane currents, remained protected. Such an active role in mediating cell death implies that vulnerability to ischemic conditions is directly proportional to the Panx1 expression pattern across inner retinal cell types (Fig. 1). Indeed, ACs are well known to be relatively more resistant to glaucoma than RGCs. In the literature, another line of evidence supporting this correlation is provided by studies where direct cytotoxicity was induced by acute conversion of Panx1 channels to a fully open state by either caspase cleavage^9,12,55,60^ or by light in an engineered optogenetic Opto-Panx1 construct^61,62^.

Extracellular ATP, released from mechanically stressed^43,45^ or dying cells during ischemic^63^ and ocular hypertensive conditions^43,45^, exerts cytotoxicity through the activation of P2X receptors and Panx1 channels^3,64^. Cytotoxic levels may also be achieved locally through the contribution of Panx1-mediated ATP release, which can trigger activation of low-affinity P2X7 receptors in either a paracrine or autocrine^65^ manner. As an example, Panx1-mediated eATP elevation in the retina, triggered by mechanical stress^43^, such as IOP spikes, led to neuronal death in the GCL of rat retinas^66^. Importantly, cytotoxic activation of P2X7 only occurs at pathologically high (>1 mM) eATP levels but not in the physiological range (<100 µM), which only causes cell permeation to small molecules and transient elevation in intracellular Ca^2+^ in primary retinal ganglion cells^67^. Consistent with the proposed mechanism, blockade of either the P2X7 receptor^68^ or enzymatic degradation of eATP by apyrase treatment also suppresses this toxicity^68^.

Taken together, our results fit a hypothetical model in which the accelerated death of Panx1-enriched RGCs, including the OFF-type cells, via over-activation of the channel represents a potential new mechanism that contributes to the selective loss of electrically active neurons in ischemic and glaucomatous pathologies. This model requires further validation but is not in conflict with the existing paradigm stating that RGCs are very sensitive to ischemia-induced mitochondrial insufficiency due to high energy dependence (reviewed in^69^). Post-ischemic metabolic failure, leading to RGC death through intracellular calcium overload, membrane depolarization and loss of ATP production, has been shown to be mechanistically mediated by Panx1 in hippocampal pyramidal neurons and RGCs ^49,52,70^, including by this study. Alternative neuronal death mechanisms involving Panx1, including those mediated by the inflammasome^71,72^, are also a possibility and will be investigated in a separate study.

In conclusion, our findings show that Panx1 plays an essential physiological role in RGCs by contributing to the electrical output of these cells. Our results also suggest that OFF-type RGCs exhibit particularly high Panx1 channel activity, which can also underlie their susceptibility to glaucoma. Importantly, the neuroprotective effect of transient Panx1 blockade provides feasibility for the pharmacological modulation of this channel as a part of a prophylactic or therapeutic intervention in acute and chronic ocular hypertension injury, including glaucoma.

## Materials and Methods

### Animals

All experiments and post-surgical care were performed in compliance with the NIH Guide for the Care and Use of Laboratory Animals and according to the University of Miami Institutional Animal Care and Use Committee approved protocols #09-037 and #15-031. Mice were bred in the University of Miami animal facility, and housed under standard conditions of temperature and humidity with a 12-h light/dark cycle and free access to food and water. Wild type (WT) animals were in the C57BL/6 background. The Panx1^fl/fl^ mouse line with LoxP sites flanking exons 3 and 4 in a single-copy Panx1 gene (B6;129-Casp4^del^ Panx1^tm1Vshe^/J)^49^ were used to generate zygotic and conditional knockout lines. These mice were backcrossed to a C57BL/6 background for at least seven generations prior to the experiments. For complete knockout mice, Panx1^fl/fl^ animals were crossed with CMV-Cre driver mice (B6.C-Tg(CMV-cre)1Cgn/J). Knockout mice with neuron-specific conditional (Thy1-Cre/Panx1) inactivation of Panx1 were generated by crossing with FVB/N-Tg(Thy1-cre)1Vln/J mice, as previously described^49^. For RGC-selective deletion of the gene, Panx1^fl/fl^ mice received intraocular injection of a replication-deficient AAV2 virus carrying a CRE expression cassette (AAV2-Cre/GFP, Vector Biolabs, Philadelphia, PA), which transduced approximately 85–90% of the RGCs with only minimal transduction of some Müller cells (Schmitt *et al*.^33^). Mouse strains with full ablation of Casp11 on C57Bl/6 background (referred to as Casp11^−/−^, Jaxx strain B6.129S4(D2)-Casp4^tm1Yuan/J^) possessing wild type Panx1, were purchased from Jackson Laboratory (Bar Harbour, ME). An alternative strain of mice with full zygotic Panx1 ablation (Panx1^−/−^/B6), developed by V. Dixit on the B6 genetic background^35^, were obtained from Genetech Inc. (Oceanside, CA) and backcrossed with C57Bl/6J for 5 generations.

### Cell culture

Neuroblastoma N2a (N2a, ATCC strain catalogue #CCL-131) cells were cultured at 37 °C and 5% CO_2_ in IMDM + GlutaMAX (Life Technologies), supplemented with 10% fetal calf serum and penicillin/ streptomycin antibiotic. N2a cells were stably transfected with pPanx1- IRES2-EGFP or pEGFP (Invitrogen) constructs using Lipofectamine 2000 (Invitrogen) with 0.6 mg/ml G418 for selection. The resulting N2a clones expressed either Panx1 (N2a-Panx1-C1 and Panx1-C3) and EGFP to label Panx1-expressing cells or EGFP alone (N2a-EGFP-B2 and N2a-EGFP-B3) in control cell lines.

### *In situ* RNA hybridization

*In situ* RNA hybridization was performed using RNAscope technology (Advanced Cell Diagnostics, Hayward, California) following the manufacturer’s protocol. Briefly, formalin fixed paraffin embedded total mouse eyes were cut into 5 μm sections and mounted on SuperFrost Plus glass slides. After de-paraffinization, slides were treated for 15 min with boiling Pretreat 2 solution, followed by pretreatment 3 (protease) for 30 min at 37 °C. To reduce chromosomal DNA background, we introduced a DNase treatment step: slides were washes 5× with water, and treated with DNase I (50 u/ml in 1× DNase I buffer, Ambion) for 40 min at 37 °C. To demonstrate that the signal comes from hybridization of probes with mRNA, some slides were treated with a mixture of DNase I and RNase A (5 mg/ml). Following 5× wash with water, slides were hybridized with RNAscope probes for 2 h at 40 °C. The fluorescent signal was visualized and captured using either Nikon Eclipse TE2000-U open-field or Leica SP2 confocal microscopes.

### Whole-cell patch clamp recordings in intact retina

Freshly dissected retinas were attached photoreceptor-side down to a translucent Millicell filter ring (Millipore, Bedford, MA), and placed on a recording chamber of an upright Nikon FN1 microscope equipped with Hoffman modulation contrast optics (Modulation Optics, Inc., Greenvale, NY). The chamber was constantly bathed (1 ml/min) with bicarbonate-buffered Ames solution (Sigma, St. Louis, MO), and equilibrated with 95% O_2_ and 5% CO_2_ at 32 °C; solutions were applied using an eight-channel superfusion system (Warner Instruments, Hamden, CT). The intracellular solution contained (in mM): 120.0 Cs-gluconate, 10.0 tetraethyl-ammonium chloride (TEA-Cl), 1.0 CaCl_2_, 1.0 MgCl_2_, 11.0 ethylene glycol-bis(beta-aminoethyl ether)-N,N,N′,N′-tetraacetic acid (EGTA), 10.0 sodium N-2-hydroxyethylpiperazine-N′-2-ethanesulfonic acid (Na-HEPES), 2.5 QX314 (an anaesthetic to block postsynaptic sodium action potentials), and 0.05% sulforhodamine B adjusted to pH 7.2 with CsOH. The calculated E_Cl_ was −58 mV. Whole-cell patch clamp recordings where performed as previously described^73,74^. A modified preconditioning protocol^31^ was applied to assess Panx1 channel activity. Cells were clamped at a −60 mV holding potential, and 250 ms steps from −80 mV to +60 mV in 10 mV increments were applied followed by three 10 s preconditioning depolarizing voltage ramps ranging from −80 mV to +60 mV. After the third ramp, the voltage steps were repeated, and only these data were used for analyses. To stimulate Panx1 channel activity, we used an extracellular “Panx1 agonist mix” solution with increased ATP and K^+^ (20 mM K^+^, 0.1 mM ATP), and lower Ca^2+^ (0.5 mM) concentrations (all from Sigma). For specific inhibition of Panx1-mediated currents, 100 µM of ^10^panx1 peptide (Anaspec, Fremont, CA), 50 nM mefloquine (MFQ; Bioblocks, San Diego, CA), 25 µM water-soluble carbenoxolone (CBX, Invitrogen), or 300 µM water-soluble probenecid (Thermo Fisher Scientific) were added to the external solution according to Ma *et al*.^75^. Data analyses was performed using Signal software (Cambridge Electronic Design, UK). Whole-cell currents were measured manually at the end of the step pulse. Patch clamp data with a duration less than 10 min were discarded.

For morphological identification, RGCs were filled with Alexa 568 during recording and confocal z-stack projections of recorded RGCs were generated immediately after. RGC cells were identified by the presence of axon and classified into three physiological classes based on 1) stratification level of their dendritic tree in ON and/or OFF inner plexiform layers of the sublamina and 2) spiking activity in response to the light stimulus. Pannexin1-activated current was recorded in whole-cell patch clamp mode using triple-ramp paradigm in response to the step pulse from −80 to 60 mV with holding potential −60 mV in control, in the presence of Panx1 of agonist mix or antagonists.

### Whole-cell patch clamp recordings from N2a cells

Recordings from stably transfected N2a cells were performed on coverslips as described above. The external solution contained (in mM): 102.0 NaCl, 10.0 D-glucose, 2.6 KCl, 1.0 CaCl_2_, 1.0 MgCl_2_ and 28.0 NaHCO_3_ adjusted with NaOH to pH 7.4. The pipette solution contained (in mM): 125.0 K gluconate, 10.0. KCl, 10.0 HEPES, 0.5 CaCl_2_, 0.5 MgCl_2_, and 0.4 EGTA adjusted with KOH to pH 7.2. These concentrations resulted in a free Ca^2+^ concentration of ~100 µM, which enhance Panx1 channel activity.

### *In vivo* retinal electrophysiology

An optimized protocol for mouse PERG recording was previously described^20,32,76^. Briefly, electrical signals were recorded in anesthetized mice from the corneal electrodes under conditions that maximized PERG amplitude (pattern stimuli of horizontal bars with a spatial frequency 0.05 cycles/deg, temporal frequency 1 Hz, contrast 100%) with substantial averaging (1800 sweeps). The PERG signal-to-noise ratio was of the order of 10:1, and the test–retest variability was of the order of 30%^20^. Balanced salt solution (BSS) drops were applied to maintain cornea transparency.

### Electrophysiology data analyses

Statistical analyses of whole-cell patch clamp recordings in the intact retina were performed using Excel 2011 (Microsoft Corporation, Redmont, WA) and OriginPro 7 (OriginLab Corporation, Northampton, MA). Plotted data are given as means ± SEM. Statistical differences of the data were evaluated by analysis of variance (ANOVA); the level of significance was set at P < 0.05. Statistical analyses of patch clamp data obtained from N2a cells was performed with Prism 5 (GraphPad Software Inc., La Jolla, CA). Comparisons between two datasets were made using the Mann-Whitney rank sum test. Comparison between three or more groups have been done with the nonparametric Kruskal-Wallis test followed by the Dunn’s Multiple Comparison post test. Statistical analysis of PERG measurements was carried out with the Mann–Whitney test. The overall level of significance was set at P < 0.05.

### AAV2 virus construct injection

AAV2-EGFP-Cre viral particles were injected under ketamine-xylasine anesthesia unilaterally into the left (experimental) eye of experimental animals. The total volume of 1.5 µL was delivered slowly into the vitreous over 30 s period to prevent acute intraocular pressure elevation; care was taken to avoid leaks, lens damage and infection (triple antibiotic ointment). Mice were analyzed by PERG recordings at 4,6 and 8 weeks prior to termination to allow for Cre-deletion and pre-existing protein elimination. Only high titer stocks, containing 5.0xE^12^-1.2-E^13^ capsids were used to ensure >90% *in vivo* infection rate. Contralateral control eye received AAV2-EGFP construct to normalize for potential the off-target effects of AAV2 virus infection. EGFP expression in control eye was assessed after termination of the series at 8 weeks to assess the efficacy of AAV2 infection.

### Behavioral test of visual acuity

Visual thresholds in experimental and control eyes were evaluated using the custom-made Optokinetic Head-Tracking system. The systems is equipped with four optokinetic monitors to display of rotating sinusoidal gratings of alternating white and black stripes, following the method of Prusky^77^. The rotation direction was changed every 30 s for a total of 6 changes per stripe thickness. Stripe thickness of the gratings was decreased stepwise by a factor of 2 until the animal could no longer track the direction of movement of the gratings. Mice were placed on a raised platform in the center of a chamber and were tracked for head movements that followed the direction of stripe movement, to score their vision. Visual acuity was defined as the highest spatial frequency yielding a response from the mouse, which was derived from the angular frequency of the stripe rotation. Data analysis included calculation of mean cycles/degree values ± SDEV.

### IOP-induced retinal ischemia-reperfusion model

Under ketamine/xylazine (80/16 mg/kg) anesthesia, pupils were dilated with 1% tropicamide and 2.5% phenylephrine hydrochloride (NutraMax Products, Inc., Gloucester, MA), and 1 drop of 0.5% proparacaine HCl (Bausch & Lomb Pharmaceuticals, Rochester, NY) was applied for corneal analgesia. Retinal ischemia was induced by an IOP increase above cystolic blood pressure (to 120 mm Hg) for 60 min by direct cannulation of the anterior chamber with a 29G needle connected to a normal saline (0.9% NaCl)-filled reservoir raised above the animal, as described in^49^). The contralateral eyes, cannulated at normal IOP, served as normotensive controls. Complete retinal ischemia, observed as the whitening of the anterior segment and blanching of the arteries, was verified microscopically. Mice were euthanized and retinas were dissected out, fixed and processed at 7 days after reperfusion. Probenecid was injected intraperitoneally 1 h prior to IOP elevation at 2.0 mM in sterile PBS according to Mawhinney *et al*.^78^.

### RGC loss assessment

Data analysis on RGC loss was performed in flat-mounted retinas sampled at 7 days post-reperfusion and immunostained with NeuN antibodies. Confocal image slices collected for depth (0–30 µm) were collapsed to generate maximum projections, and used for RGC counts with MetaMorph software (Universal Imaging Co., Bedford Hills, NY) after thresholding and manual exclusion of artifacts. Retinas were sampled from 20 fields in 4 retinal quadrants, each samples in 3 regions of the same eccentricities (0.5 mm, 1.0 mm, 1.5 mm from the optic disk) as previously described^49^. RGC loss was calculated as a percentage of NeuN-positive cells in experimental eyes relative to sham-operated contralateral control eyes. The cell density data (n ≥ 5) were averaged for each group/genotype; statistical analyses data were analyzed with one-way ANOVA followed by Tukey test for multiple comparisons; P values ≤ 0.05 were considered statistically significant.

### Oxygen/glucose deprivation model

N2a cells were incubated for 4 h in a hypoxic chamber (BioSpherix Ltd., Parish, NY) using glucose-free oxygen/glucose deprivation (OGD) solution (Hank’s BSS [HBSS] with sucrose, deoxygenated by bubbling with N_2_ for 1 h at 37 °C) as described in Dvoriantchikova *et al*.^49^. Subsequently, the culture medium was changed to normoxic Neurobasal/B27 media for 1–24 h in a 5% CO_2_ atmosphere (“reperfusion” phase) prior to the analysis.

### Statistical analysis

Real-time PCR and cell density data were analyzed for significance with one-way ANOVA followed by Tukey test for multiple comparisons. For single comparisons, Student’s t test was applied. P values ≤ 0.05 were considered statistically significant.

The description of common methods, immunohistochemistry, isolation of primary retinal cells, including real-time PCR, Western blot analysis, flash electroretinogram recordings, neuronal death assay in cultured N2a is provided in the Supplementary information.

## Electronic supplementary material

Supplementary information

Supplementary data 1
