# Supplementary material for: Pannexin 1 sustains the electrophysiological responsiveness of retinal ganglion cells
Source: Sci Rep. 2018 Apr 11;8:5797. doi: 10.1038/s41598-018-23894-2 (PMC5895610; doi:10.1038/s41598-018-23894-2)
Supplement: Supplementary file 1 — Supplementary information [file 41598_2018_23894_MOESM1_ESM.doc]

**Pannexin1 sustains electrophysiological responsiveness of retinal ganglion cells**

Galina Dvoriantchikova^1^, Alexey Pronin^2^, Sarah Kurtenbach^1^, Abduqodir Toychiev^3^, Tsung-Han Chou^1^, Christopher W. Yee^4^, Breanne Prindeville^1^, Junior Tayou^2^, Vittorio Porciatti^1^, Botir T. Sagdullaev^3,4^, Vladlen Z. Slepak^2^, Valery I. Shestopalov^1,5,6^**^*^**

**Supplementary Information***.*

The online version of this article contains supplementary material, supporting the conclusions in this article:

**Supplementary Methods**

**Immunohistochemistry**

Eyes were enucleated, fixed in 4% paraformaldehyde for 1 h, and cryoprotected with 30% sucrose. Retinas were sectioned to a thickness of 80 µm on a vibratome (Vibratome, St. Louis, MO) and incubated with a primary antibody for 4–16 h. Retinal flat-mounts were incubated with primary antibodies for 3–5 days at 4ºC to ensure even staining . To identify Panx1, we used C-terminal rabbit polyclonal antibodies against mouse Panx1 (CT-395, 1:1,000-3,000 dilution, provided by D. Liard and S. Penuela^1^); to identify RGCs, we used anti-neuronal Class III β-Tubulin (clone TUJ1, Covance, Princeton, NJ, 1:500 dilution); Alexa 488-conjugated anti-NeuN (MAB377X, 1:200 dilution; EMD Millipore, Billerica, MA), and anti-Brn3A (Santa Cruz Biotechnology (Dallas, TX) clone 14A6, 1:300 dilution). Secondary AlexaFluor dye-labeled antibodies (Thermo Fisher Scientific, Waltham, MA) were applied for imaging with the Leica TSL AOBS SP5 confocal microscope (Leica Microsystems, Wetzlar, Germany); controls with primary antibodies omitted were used for specificity tests.

**Isolation of primary retinal cells**

RGCs were isolated from P5- to P7-old pups according to the modified two-step immunopanning method, described previously^2^. Primary purified RGCs were grown in Neurobasal/B27 media (Thermo Fisher Life Technologies, Grand Island, NY). Muller glia were prepared as previously described ^3^. Primary Muller glia were cultured in Dulbecco’s Modified Eagle’s Medium (DMEM; Life Technologies, Grand Island, NY) containing 10% heat-inactivated fetal bovine serum (FBS; Life Technologies) and 1% antibiotic-antimycotic (Life Technologies). After growing cells to confluence, the flask was shaken overnight on an orbital shaker for 16 h at 37°C, and non-attached cells were removed.

**Real-time PCR analysis**

Gene expression was assessed by real-time PCR, using gene-specific primer pairs Px1-F: CAAGGGAGAGGACCAGGGC and Px1-R: ATCTATTCTTCTATGACGCTG, as previously described ^4^. For the quantitative PCR this pair of primers was validated to span an intron and to amplify only one product. Total RNA was extracted using the Absolutely RNA Nanoprep kit (Agilent Technologies, Wilmington, DE) and reverse transcribed with the Reverse Transcription System (Promega, Fitchburg, WI, USA). Real-time PCR was performed in the Rotor-Gene 6000 Cycler (Corbett Research, Mortlake, Australia) using the SYBR GREEN PCR MasterMix (Qiagen, Valencia, CA). Relative expression was calculated by comparison with a standard curve following normalization to the β-actin (Actb) gene.

**Flash electroretinogram recordings**

A light-adapted flash electroretinogram (FERG), an index of outer retinal function, was recorded with undilated pupils to match the PERG recordings. Uniform stimuli for FERG recordings consisted of strobe flash stimuli of 20 cd/m^2^ per second superimposed on a steady background light (12 cd/m^2^), which was presented within a Ganzfeld bowl. Under these conditions, rod activity is largely suppressed, while cone activity is minimally suppressed ^5^. Data were primarily processed using a simple macro written in Sigmaplot (version 11.2, Systat Software Inc., San Jose, CA; ^6^). Subsequently, data were processed using Excel 2011 (Microsoft Corporation). Statistical analyses were performed with Prism 5 and presented using box and whisker plots.

**Western blot analysis**

N2a cells were homogenized in 1x RIPA lysis buffer (Thermo Scientific, Inc.) supplemented with complete protease inhibitor (Roche), after which equal amounts of protein, as measured using the BCA kit (Pierce), were resolved on SDS-PAGE gradient 4–12% Bis-Tris gels and then transferred to PVDF membranes (Invitrogen). Membranes were probed with primary antibody overnight, washed in 0.15% Tween 20 in TBS, and incubated for 1 h with secondary antibody (1:1,000, Amersham Biosciences, NJ). Anti β-actin antibody (clone AC-15, 1:1000 dilution; Abcam Inc., Eugene, OR) served as the loading control. Proteins were visualized and quantified using SuperSignal chemiluminescent substrates (Pierce Inc. Rockford, IL USA) in the FUJIFILM imaging station. Western blot quantitation data were normalized to β-actin transcript abundances and calculated as a percentage of the control (mean±SD, n=3).

**Dye uptake in cell cultures**

Dye uptake by N2a cells was measured using FLUOstar Omega plate reader (BMG Labtech). Cells were grown on clear bottom poly-L-lysine coated 96-well plates until they reach confluency. At time zero on the day of the assay the cell culture medium was removed and cells were rinsed once with HBSS (100 μL/well). Then HBSS with 20 μM ethidium bromide (USB) and the indicated compounds was added (70 μL/well). If OGD was performed, OGD medium was used for the assay instead of HBSS and the plates with cells were placed inside the OGD chamber. At indicated time points ethidium fluorescence was measured using 355 nm excitation and 620 nm emission filters. Some wells were incubated in the presence of 0.03% saponin to permeabilize cells and get the maximal dye uptake value. Values from the wells containing no cells were subtracted as the background and presented as percentages of maximal uptake (EtBr) or release (EGFP) values. Significance was calculated using one-way ANOVA followed by Tukey test for multiple comparisons.

**Neuronal death assay in N2a cultures.**

N2a cell viability was assessed by detecting cytoplasmic protein elevation in the media. Intracellular EGFP protein release (in cells expressing either Panx1-IRES-EGFP or pEGFP constructs) was measured using FLUOstar Omega plate reader (BMG Labtech). Cells were grown on clear bottom poly-L-lysine coated 96-well plates until they reach confluency. At time zero on the day of the assay the cell culture medium was removed, cells were rinsed once with HBSS (100 µL/well) and either HBSS or OGD medium with indicated compounds was added (70 µL/well). If OGD was performed, the plates with cells were placed inside the OGD chamber. At indicated time points supernatants were removed from the wells and placed into a different 96-well plate. EGFP fluorescence was measured using 485 nm excitation and 520 nm emission filters. Values from the wells containing no cells were subtracted as the background. To determine if ATP released by the cells contributed to their death, GFP release was performed in the presence of 10 u/ml apyrase from potato (Sigma). Cells death after OGD challenge was determined using the Vybrant Apoptosis Assay Kit #2 (Invitrogen) by automated cell counting with MetaMorph imaging software. The percentage of necrotic cells (Annexin V and PI) and apoptotic cells (only Annexin V) was determined in ≥10 images for each biological replicate. Data are presented as means ± SE; significance was calculated using one-way ANOVA followed by Tukey test for multiple comparisons; P≤0.05 were considered significant.

**References to Supplementary Methods.**

1 Penuela, S. *et al.* Pannexin 1 and pannexin 3 are glycoproteins that exhibit many distinct characteristics from the connexin family of gap junction proteins. *J Cell Sci* **120**, 3772-3783 (2007).

2 Barakat, D. J., Dvoriantchikova, G., Ivanov, D. & Shestopalov, V. I. Astroglial NF-kappaB mediates oxidative stress by regulation of NADPH oxidase in a model of retinal ischemia reperfusion injury. *J Neurochem* **120**, 586-597 (2012).

3 Hauck, S. M., Suppmann, S. & Ueffing, M. Proteomic profiling of primary retinal Muller glia cells reveals a shift in expression patterns upon adaptation to in vitro conditions. *Glia* **44**, 251-263 (2003).

4 Dvoriantchikova, G., Ivanov, D., Panchin, Y. & Shestopalov, V. I. Expression of pannexin family of proteins in the retina. *FEBS Lett* **580**, 2178-2182 (2006).

5 Lyubarsky, A. L., Daniele, L. L. & Pugh, E. N., Jr. From candelas to photoisomerizations in the mouse eye by rhodopsin bleaching in situ and the light-rearing dependence of the major components of the mouse ERG. *Vision research* **44**, 3235-3251 (2004).

6 Porciatti, V., Chou, T. H. & Feuer, W. J. C57BL/6J, DBA/2J, and DBA/2J.Gpnmb mice have different visual signal processing in the inner retina. *Molecular vision* **16**, 2939-2947 (2010).
