# Supplementary material for: Pannexin 1 sustains the electrophysiological responsiveness of retinal ganglion cells
Source: Sci Rep. 2018 Apr 11;8:5797. doi: 10.1038/s41598-018-23894-2 (PMC5895610; doi:10.1038/s41598-018-23894-2)
Supplement: Supplementary file 2 — Supplementary data 1 [file 41598_2018_23894_MOESM2_ESM.doc]

**Pannexin1 sustains electrophysiological responsiveness of retinal ganglion cells**

Galina Dvoriantchikova^1^, Alexey Pronin^2^, Sarah Kurtenbach^1^, Abduqodir Toychiev^3^, Tsung-Han Chou^1^, Christopher W. Yee^4^, Breanne Prindeville^1^, Junior Tayou^2^, Vittorio Porciatti^1^, Botir T. Sagdullaev^3,4^, Vladlen Z. Slepak^2^, Valery I. Shestopalov^1,5,6^**^*^**

**Supplementary Data***.*

The online version of this article contains supplementary material, supporting the conclusions in this article:

**1: Supplementary Figure S1:** Behavioral test of visual acuity using optokinetic system.

**2: Supplementary Figure S2**: Casp11 inactivation does not protect RGCs in retinal ischemia-reperfusion injury model.

**3:** **Supplementary Figure S3.** High level of Panx1 expression increased susceptibility of N2a cells to ischemia.

**4: Supplementary Figure S4**

**5. Table S1:** PERG and FERG recording data for mice injected with AAV2-GFP-CRE and AAV2-GFP constructs.

**6:** **Table S2:** Whole cell current recorded from WT retina.

**7:** **Table S3:** Whole cell current recorded from WT retina. Unresponsive cells sub-population.

**8: Table S4:** Whole cell current recorded from Panx1^-/-^ retina. All data points collected at 60 mV.

**9.** **DNA sequence file** of pPanx1-IRES-EGFP plasmid construct used to generate stable clones N2a-Panx1-EGFP-C1 and –C3.

1. **Supplementary Figure S1.**

**Behavioral test of visual acuity** using optokinetic system with head-tracking/ These tests did not detect any significant differences between control C56Bl6 (WT, white box) and germline Panx1^-/-^ (Panx1KO, red box) mice using standard 100% contrast settings. Visual acuity is calculated in cycled/degree (c/d); data are presented as means ± SE; n=10; significance calculated using Student’s t-test.

**2. Supplementary Figure S2**

**Genetic ablation of Casp11 does not protect RGCs in retinal ischemia-reperfusion injury model.** Mice with zygotic ablation of Casp11 (Casp11KO) showed RGC loss rates similar to that in wild type (WT) eyes; Panx1^-/-^ mice with intact Casp11 (Px1KO/Casp11^+^) showed a significant 35% reduction in RGC loss rates relative to WT. RGC loss was calculated as a percentage of NeuN-positive cells vs. sham-operated contralateral controls. Data are presented as means ± SD; * P<0.05; n=5-11, significance calculated using one-way ANOVA and Tukey test for multiple comparisons.

**3. Supplementary Figure S3**

**
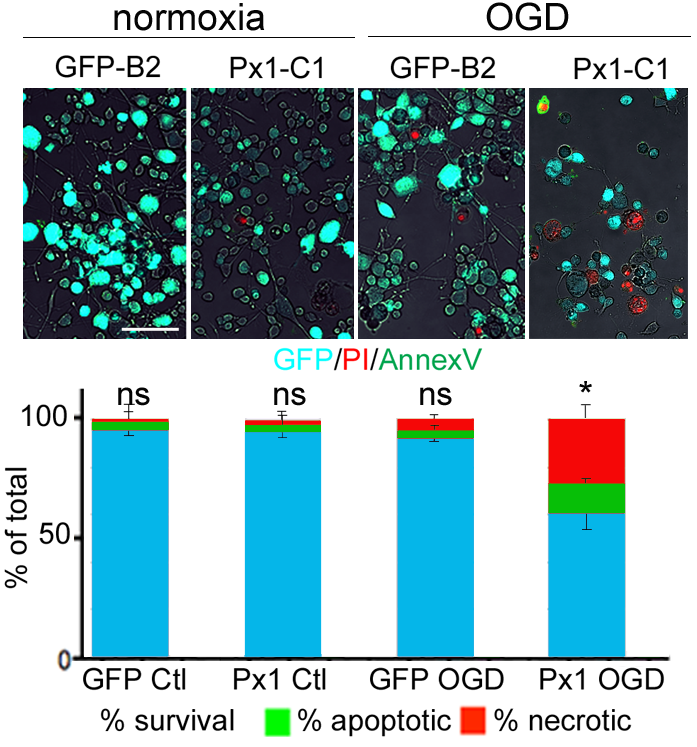
**

**High level of Panx1 expression increases susceptibility of N2a cells to ischemia.** **A**. Representative micrographs of AnnexinV (AnnexV, green) and propidium iodide (PI, red) labeling in in N2a-Panx1-C1 (Px1-C1) cells and control N2a-EGFP-B2 (GFP-B2) cell lines 24 h after exposure to normoxic or ischemic (OGD) conditions; EGFP labeling is shown in cyan. Bar, 25µm **B**. Quantitation of cell death rates, shown as percentages of total cell counts; *P<0.05; n=10, significance calculated using one-way ANOVA and Tukey test for multiple comparisons.

**4. Supplementary Figure S4**

**Original unmodified Western blot data image for Figure 5B.** The image has been captured using a digital (filmless) Odyssey® imager system and fluorescence probe labeling.

**9. Panx1 gene sequence validation.**

The cDNA, amplified by RT-PCR from murine taste bud tissue, was inserted into pIRES2-EGFP vector (NheI/EcoRI sites). This sequence corresponds to nucleotides 431-1739 of mouse Panx1 gene (database entry NM_019482.2) and was used for validation of clones N2a-Panx1-EGFP-C1 and N2a-Panx1-EGFP-C3:

gcgccttgac c**atg**gccatc gcccacttgg ccacggagta tgtgttctcg

gacttcttgc tgaaggagcc caccgagccc aagttcaagg ggctgcgact ggagctggcg

gtggacaaga tggtcacatg tattgccgtg ggtctacctc tgctgctcat ctcgctggcc

ttcgctcagg agatctccat cggtacccag ataagctgct tctccccgag ttctttctcc

tggcgacagg ctgcctttgt ggattcatac tgctgggctg ctgtacagca gaagagctcc

ctgcagagcg agtctggaaa cctcccactg tggctgcaca agttcttccc ctacatccta

ctgctgtttg ccatactcct gtacctgccc gcactcttct ggcgcttctc tgcagctcca

cacctctgct cagacctgaa gtttatcatg gaggaacttg acaaagtcta caaccgcgcc

atcaaggctg ccaagagtgc tcgagatttg gacctaagag acggacctgg acccccagga

gtgactgaga atgtggggca gagtctgtgg gagatatctg aaagccactt caagtaccca

atcgtggagc agtacttgaa gacaaaaaag aactctagtc atttaatcat gaaatacatt

agctgccggc tggtgacatt tgtggttata ctgttggcat gtatctactt gagctattac

ttcagcctct cttcactctc ggacgagttt ctgtgcagca tcaaatcagg cgtcctgaaa

aatgacagca ccatccccga tcgcttccag tgcaagctca tcgccgtggg catcttccag

ctgctcagcc tcattaacct cattgtgtat gctctgctga ttcccgtggt cgtctacacg

ttcttcatcc cattccggca gaaaacggac attctcaaag tgtatgaaat cctgcccacc

ttcgatgttc tacatttcaa gtctgaaggc tacaatgact tgagcctcta caaccttttt

ctggaagaga acataagtga gctcaaatcg tacaagtgtc tgaaggtgct ggagaacatt

aaaagcaatg ggcagggcat tgaccccatg ctactcctga caaacctggg catgattaag

atggacatca ttgatggaaa aattcccacg tccctacaga ccaagggaga ggaccagggc

agccagagag tggagttcaa agatttggac ctgagcagcg aggctgcagc aaacaatggg

gagaagaact ctcgccagag gcttctgaat ccgtcctgc**t aa**tggtttcc ttcttgaa
